# FcγRIIIb Restricts Antibody-Dependent Destruction of Cancer Cells by Human Neutrophils

**DOI:** 10.3389/fimmu.2018.03124

**Published:** 2019-01-30

**Authors:** Louise W. Treffers, Michel van Houdt, Christine W. Bruggeman, Marieke H. Heineke, Xi Wen Zhao, Joris van der Heijden, Sietse Q. Nagelkerke, Paul J. J. H. Verkuijlen, Judy Geissler, Suzanne Lissenberg-Thunnissen, Thomas Valerius, Matthias Peipp, Katka Franke, Robin van Bruggen, Taco W. Kuijpers, Marjolein van Egmond, Gestur Vidarsson, Hanke L. Matlung, Timo K. van den Berg

**Affiliations:** ^1^Sanquin Research, and Landsteiner Laboratory, Amsterdam UMC, University of Amsterdam, Amsterdam, Netherlands; ^2^Department of Molecular Cell Biology and Immunology, Amsterdam UMC, Amsterdam Infection and Immunity Institute, Vrije Universiteit Amsterdam, Amsterdam, Netherlands; ^3^Emma Children's Hospital, Amsterdam UMC, University of Amsterdam, Amsterdam, Netherlands; ^4^Division of Stem Cell Transplantation and Immunotherapy, Department of Internal Medicine II, Kiel University, Kiel, Germany

**Keywords:** FcγRIIIb, neutrophil, ADCC, cancer, granulocyte, Fc-receptor, CNV, glycoengineering

## Abstract

The function of the low-affinity IgG-receptor FcγRIIIb (CD16b), which is uniquely and abundantly expressed on human granulocytes, is not clear. Unlike the other Fcγ receptors (FcγR), it is a glycophosphatidyl inositol (GPI) -anchored molecule and does not have intracellular signaling motifs. Nevertheless, FcγRIIIb can cooperate with other FcγR to promote phagocytosis of antibody-opsonized microbes by human neutrophils. Here we have investigated the role of FcγRIIIb during antibody-dependent cellular cytotoxicity (ADCC) by neutrophils toward solid cancer cells coated with either trastuzumab (anti-HER2) or cetuximab (anti-EGFR). Inhibiting FcγRIIIb using CD16-F(ab')_2_ blocking antibodies resulted in substantially enhanced ADCC. ADCC was completely dependent on FcγRIIa (CD32a) and the enhanced ADCC seen after FcγRIIIb blockade therefore suggested that FcγRIIIb was competing with FcγRIIa for IgG on the opsonized target cells. Interestingly, the function of neutrophil FcγRIIIb as a decoy receptor was further supported by using neutrophils from individuals with different gene copy numbers of *FCGR3B* causing different levels of surface FcγRIIIb expression. Individuals with one copy of *FCGR3B* showed higher levels of ADCC compared to those with two or more copies. Finally, we show that therapeutic antibodies intended to improve FcγRIIIa (CD16a)-dependent natural killer (NK) cell ADCC due to the lack of fucosylation on the N-linked glycan at position N297 of the IgG_1_ heavy chain Fc-region, show decreased ADCC as compared to regularly fucosylated antibodies. Together, these data confirm FcγRIIIb as a negative regulator of neutrophil ADCC toward tumor cells and a potential target for enhancing tumor cell destruction by neutrophils.

## Introduction

Fc-receptors play a vital role in cancer immunotherapy by inducing ADCC and antibody dependent cellular phagocytosis (ADCP). Most cancer targeting therapeutic antibodies currently on the market are of the IgG class, and thus human FcγRs constitute the key receptors for ADCC during cancer immunotherapy (1). The principal FcγR receptor on neutrophils required for mediating ADCC of solid cancer cells appears to be FcγRIIa (2, 3), with ~30–60-thousand copies expressed per cell (4), sometimes in combination with the activating receptor FcγRIIc, present on a minority of about 15–20% of Caucasian individuals (5). The high affinity receptor FcγRI (CD64) is only present on activated neutrophils, but does generally not contribute to ADCC of solid cancer cells even when expressed (3). Both FcγRI and FcγRIIa signal via immunoreceptor tyrosine-based activation motifs (ITAM), encoded in the cytoplasmic tail of the receptors (FcγRIIa) or in the associated γ-chain (FcγRI). Lastly, neutrophils express the highly abundant, 100–200-thousand copies per cell, low affinity receptor FcγRIIIb, which is a GPI-linked Fc-receptor that lacks intrinsic intracellular signaling capacity (4). This receptor is selectively present on neutrophils and on a subset of basophils (6). In spite of the lack for direct signaling through FcγRIIIb evidence from a number of studies show that FcγRIIIb cooperates together with other FcγR in the context of the phagocytosis of opsonized microbes (7). This suggests that the abundantly expressed FcγRIIIb primarily acts to facilitate enhanced recognition and that ITAM signaling via the other FcγR, in particular FcγRIIa, is sufficient, or at least instrumental, to trigger the phagocytic process. The FcγRIIIb-encoding gene, *FCGR3B*, which only occurs in humans and certain primates (8), is located within the *FCGR2/3* locus on human chromosome 1, where it is prone to gene copy number variation (CNV) (9). The CNV of *FCGR3B* ranges from very rare individuals with no *FCGR3B*, to individuals with five copies of this gene (10). *FCGR3B* CNV has been shown to affect various diseases, i.e., a low CNV of *FCGR3B* was shown to result in an increased susceptibility to autoimmune diseases like systemic lupus erythematosus (SLE) (11, 12), primary Sjogren's syndrome (pSS) (12), Wegener's granulomatosis (WG) (12) and rheumatoid arthritis (RA) (13). A high CNV of *FCGR3B* has been associated with psoriasis vulgaris in Han Chinese (14). Nevertheless, no enhanced susceptibility to bacterial or fungal infection was observed in very rare individuals lacking FcγRIIIb expression (15), also showing that their neutrophils were able to function normally in regards to phagocytosis and superoxide generation (16). In addition, several polymorphic variants of the *FCGR3B* gene, known as the NA1, NA2, and SH haplotypes exist (17, 18), which do not result in marked differences in IgG-affinity. On the level of neutrophil-mediated ADCC of cancer cells all polymorphic variants appear similarly effective (3), but neutrophils from NA1NA1 individuals have been reported to bind and phagocytose IgG-opsonized bacteria and red cells somewhat more effectively than their heterozygous NA1NA2 and homozygous NA2NA2 counterparts (19, 20).

Neutrophils constitute a major first line of host immune defense against fungal and bacterial infection (21). After extravasation from blood circulation they can enter a variety of tissues, including solid tumors (22–25). And even though the role of neutrophils in cancer is complex, with evidence for both positive or negative effects on tumor development (26), it is clear that neutrophils can contribute to the destruction of cancer cells particularly upon treatment with cancer therapeutic antibodies, as demonstrated now in a variety of animal models (27–30). Recently, we have found that neutrophils destroy antibody-opsonized cancer cells by a unique cytotoxic mechanism, termed *trogoptosis*, where neutrophils take up small pieces of cancer cell membrane, which leads to mechanical injury of the plasma membrane of cancer cells causing necrotic cell death (31). This neutrophil-mediated cytotoxic process can further be enhanced by inhibiting the interaction between the innate inhibitory immunoreceptor signal regulatory protein α (SIRPα) and CD47 (31–33). SIRPα is specifically expressed on myeloid cells and interacts with its ligand CD47, which is expressed ubiquitously, and is often overexpressed on cancer cells, acting as a “don't eat me” signal to prevent phagocytosis by macrophages (33–35). Interference with CD47-SIRPα interactions has also been shown to increase ADCC by monocytes and neutrophils, making this interaction an innate immune checkpoint and an attractive target for enhancing antibody therapy in cancer (32, 33, 36). Obviously, it is of interest to identify other pathways that negatively impact neutrophil ADCC.

Even though FcγRIIIb is a very abundant protein on neutrophils (37), its actual function has remained uncertain. Available evidence in the context of phagocytosis of antibody-opsonized bacteria by human neutrophils suggests that FcγRIIIb cooperates with activating FcγR, like FcγRIIa/c, to promote phagocytosis (7, 38–40), and we have confirmed this in the current study. However, here we show that with respect to neutrophil mediated ADCC, FcγRIIIb rather acts as a decoy receptor for IgG, likewise competing with FcγRIIa for the binding of therapeutic antibodies, thereby resulting in decreased ADCC. Thus, in the context of cancer FcγRIIIb on neutrophils uniquely functions as a limiting factor, thereby identifying it a as potential target for enhancing the therapeutic efficacy of cancer therapeutic antibodies.

## Materials and Methods

### Cells and Culture

The HER2/Neu-positive human breast cancer carcinoma cell line SKBR3 (ATCC) was cultured in IMDM medium (Gibco) supplemented with 20% fetal bovine serum, 2 mM L-glutamine, 100 U/mL penicillin and 100 μg/mL streptomycin at 37°C and 5% CO_2_. SKBR3-CD47KD cells were generated by lentiviral transduction of pLKO.1-puro—CD47KD (5′ ccgggcacaattacttggactagttctcgagaactagtccaagtaattgtgcttttt 3′), resulting in a CD47 expression of 10–15% of the parental cell line according to instructions provided by the manufacturer (Sigma), as show previously (32). Transduced cells were selected with 1 μg/mL of puromycin. As control cell line, empty vector shRNA were used (SKBR3-SCR). The CD47 knockdown cell line was routinely verified by flow cytometry.

The EGFR-positive human epidermoid carcinoma cell line A431 (ATCC) was cultured in RPMI medium (Gibco) supplemented with 10% fetal bovine serum, 2 mM L-glutamine, 100 U/mL penicillin and 100 μg/mL streptomycin at 37°C and 5% CO_2_. A431-CD47KO cell lines were generated by lentiviral transduction of pLentiCrispR-v2—CD47KO [pLentiCrispR-v2 was a gift from Feng Zhang (Addgene plasmid #52961)], using 5′ cagcaacagcgccgctacca 3′ as the CD47 CrispR target sequence. Transduced cells were selected with 1 μg/mL of puromycin, followed by limiting dilution. A clone lacking CD47 expression was selected by flow cytometry. An A431-SCR cell line was used as control for the CD47KO generated using CRISPR-Cas9 technology using a scrambled vector.

### Neutrophil Isolation

Neutrophils from healthy donors were isolated as previously described (41). In short, granulocytes were isolated from blood by density gradient centrifugation (2,000 rpm, 20 min, 20°C) with isotonic Percoll (1.069 g/mL) and erythrocyte lysis. The pellet fraction was lysed with ice-cold NH4Cl (155 mmol/LNH,CI, 10 mmol/L KHCO, 0.1 mmol/L EDTA, pH 7.4) solution for 5–10 min to destroy erythrocytes. Cells were centrifuged at 4°C (1,500 rpm, 5 min), and residual erythrocytes were lysed for another 5 min. After this, granulocytes were washed twice in cold phosphate buffered saline (PBS) containing HSA (0.5% wt/vol).Isolated neutrophils were used at a concentration of 5 × 10^6^ cells/mL. Cells were cultured in HEPES^+^ medium (containing 132 mM NaCl, 6.0 mM KCl, 1.0 mM CaCl_2_, 1.0 mM MgSO_4_, 1.2 mM K_2_HPO_4_, 20 mM Hepes, 5.5 mM glucose, and 0.5% HSA), in the presence of 10 ng/ml clinical grade G-CSF (Neupogen; Amgen, Breda, The Netherlands) and 50 ng/mL recombinant human interferon-γ (Pepro Tech Inc, USA) at a concentration of 5 × 10^6^ cells/mL for 4 or 16 h. After 16 h, cell viability was determined by the percentage of FITC-Annexin V (BD Pharmingen, San Diego, CA) positive cells on FACS, after which the cell concentration was corrected to 5 × 10^6^ viable cells/mL. Cells were consequently washed and prepared for analysis by ADCC assay. All blood was obtained after informed consent and according to the Declaration of Helsinki principles (version Seoul 2008).

### Antibodies and Reagents

FcγR expression was determined on FACS and depicted as MFI (median fluorescent intensity) using the following antibodies: anti-human FcγRI (Clone 10.1, mouse IgG1, BD Pharmingen, San Diego, CA), anti-human FcγRIIa (Clone AT10, mouse IgG1, AbD Serotec, Oxford, U.K.), anti-human FcγRIIIb (Clone 3G8, mouse IgG1, BD Pharmingen, San Diego, CA), all FITC labeled. FcγRs antagonistic antibodies were used in ADCC and trogocytosis assays at a final concentration of 5 μg/mL: monovalent human Fc fragments (Bethyl, USA) for blocking FcγRI as used previously (3), anti-human CD32 F(ab')_2_ (Clone 7.3, Ancell) to block FcγRIIa/b/c, anti-human CD16 F(ab')_2_ (Clone 3G8, Ancell) to block FcγRIIIa/b at a concentration of 10 μg/mL. CD11b expression was determined with the FITC labeled anti-CD11b antibody (Lot 8000236273, Pelicluster), and SIRPα with the FITC labeled mouse IgG_1_ antibody 12C4, previously described in (32). FITC-labeled mouse IgG1 was used as isotype control (Pelicluster). Afucosylated trastuzumab was generated in our laboratory as described before for afucosylated rituximab (42). Briefly, CHO-KI or Lec13 cells were transfected with antibody LC and HC expression constructs using transfection kit V from the Amaxa Nucleofectior System (Lonza, Cologne, Germany). The medium was exchanged by culture medium after 48 h, which contained 500 μg/mL hygromycin B. Single-cell subclones were created by limiting dilution. The produced antibodies were purified from the cell culture supernatant using CaptureSelect^TM^ IgG-CH1 Affinity Matrix (Thermo Fisher Scientific). IgA2-HER2 was generated by synthesizing (IDT, Leuven, Belgium) the variable heavy and light chain V gene encoding for trastuzumab (sequence as obtained from https://www.drugbank.ca/) and cloning into pcDNA3.1 expression vectors encoding for the constant regions for IgA2 and kappa, respectively, as described previously (43). The resulting expression vectors were then used to produce the antibodies in HEK Freestyle cells as we described previously (44, 45). Briefly, after transfection, cell supernatant was harvested after 5 days, after which cells were centrifuged (≥4000 g) and the supernatant was filtered using a 0.45 nm puradisc syringe filter (Whatmann, GE Healthcare, 10462100). Antibody concentration was determined via enzyme-linked immunosorbert assay (ELISA), as described previously (46). To create afucosylated antibodies, the decoy substrates for fucosylation, 2-deoxy-2-fluoro-l-fucose (2FF) (Carbosynth, MD06089) were added 4 h post transfection. Similarly, human anti-pneumococcal serotype 6B Gdob1 antibodies (IgG_1_) (47, 48), regular and afucosylated were produced in the same system (44, 45). They were used at a concentration of 10 μg/mL throughout the experiment to opsonize *S. pneumoniae*. Polyclonal human IgG (IVIG, nanogam, Sanquin) was used to opsonize *S. aureus* at a concentration of 1 mg/mL) for 10 min at 37°C.

### ADCC

Cancer cell lines were labeled with 100 μCi ^51^Cr (Perkin-Elmer) for 90 min at 37°C. After 3 washes with PBS, 5 × 10^3^ cells were incubated in RPMI medium supplemented with 10% fetal bovine serum, 2 mM L-glutamine, 100 U/mL penicillin and 100 μg/mL streptomycin for 4 h at 37°C and 5% CO_2_ in a 96-wells U-bottom plate together with neutrophils in a E:T ratio of 50:1 in the presence of 5 μg/mL therapeutic antibody. After the incubation supernatant was harvested and analyzed for radioactivity using a gamma counter (Wallac). The percentage of cytotoxicity was calculated as [(experimental cpm- spontaneous cpm)/ (total cpm–spontaneous cpm)] × 100%. All conditions were measured in triplicate.

### Trogocytosis Assay

To determine the amount of tumor membrane taken up by neutrophils a FACS based assay was used. Cancer cells were labeled with a lipophilic membrane dye (DiO, 5 μM, Invitrogen) for 30 min at 37°C. After washing the target cells with PBS they were incubated with neutrophils in a U-bottom 96-wells plate at a E:T ratio of 5:1 in the absence or presence of 0.5 μg/mL therapeutic antibody. Samples were fixed with stopbuffer containing 0.5% PFA, 1% BSA and 20 mM NaF and measured by flow cytometry. After gating for neutrophil population, the mean fluorescent intensity (MFI) and the percentage of cells positive for DiO were determined.

### Bacterial Phagogytosis

Uptake of FITC labeled *S. aureus* was performed in a 96 wells plate for 15 min at 37°C shaking, with 0.5 × 10^6^ neutrophils and 25 × 10^6^ bacteria in a final volume of 250 μL in HEPES^+^ medium. Bacteria were opsonized with polyclonal IgG (IVIG) (1 mg/mL) for 10 min at 37°C. Cells were fixed with stopbuffer (0.5% PFA, 1% BSA, 20 mM NaF) for 30 min at 4°C and measured by flow cytometry (BD FACSCanto II). Uptake of Dy488 labeled heat killed *Streptococcus Pneumoniae* was performed in a 96 wells plate for 30 min at 37°C while shaking, with 1.5 × 10^4^ neutrophils and 5 × 10^6^ bacteria in a final volume of 225 μL in HEPES^+^ medium. When applicable, neutrophils were incubated with FcyR blockers for 15 min at RT. Bacteria were opsonized with GDob1 antibody at a concentration of 10 μg/mL throughout the experiment. Cells were fixed with stopbuffer (0.5% PFA, 1% BSA, 20 mM NaF) for 30 min at 4°C and measured by flow cytometry (BD FACSCanto II).

### MLPA

Genotyping of individuals for *FCGR3B* CNV was performed using the *FCGR*-specific Multiplex Ligation-dependent Probe Amplification (MLPA) assay (MRC Holland), using genomic DNA isolated from whole blood with the QIAamp® kit (Qiagen, Hilden, Germany). The MLPA assay was performed as described previously (49). In brief, 5 μL of DNA (20 ng/μL) was denatured at 98°C for 5 min and subsequently cooled to 25°C in a thermal cycler with heated lid; To each sample 1.5 μL buffer and 1.5 μL buffer probe mix were added and incubated for 1 min at 95°C, followed by 16 h at 60°C. After this, 32 μL of ligase-65 mix was added to each sample at 54°C, followed by an incubation of 15 min at 54°C and 5 min at 98°C, followed by a 4 times dilution of the ligation mixture. This was followed by addition of 10 μL of polymerase mix, which contained one single primer pair, after which the polymerase chain reaction (PCR) was started immediately. PCR conditions were 36 cycles of 30 s at 95°C, 30 s at 60°C, and 60 s at 72°C, followed by 20 min at 72°C. After the PCR reaction, 1 μL of the PCR reaction was mixed with 0.5 μL CXR 60–400 (Promega, Madison, WI) internal size standards and 8.5 μL deionized formamide, and the mixture was incubated for 10 min at 90°C. The products were then separated by electrophoresis on an ABI-3130XL (Applied Biosystems, Foster City, CA).Data were analyzed using GeneMarker v1.6 sofware.

### IL-8 ELISA

IL-8 production was measured using the Human IL-8 ELISA Ready-SET-Go! (2nd Generation) kit (eBioscience, Thermo Fisher Scientific, Waltham, MA) according to manufacturer's instructions. Wavelengths were measured with an iMark microplate absorbance reader (Bio-rad Laboratories, Hercules, CA).

### Study Approval

The study was performed according to national regulations with respect to the use of human materials from healthy, anonymized volunteers with written informed consent, and the experiments were approved by the Medical Ethical Committee of the Academic Medical Center in Amsterdam according to the Declaration of Helsinki principles (version Seoul 2008).

### Data Analysis and Statistics

Statistical differences were determined by either paired or ordinary one way ANOVA, with Sidak or Dunnett's *post-test*, or by paired student's *t*-test, as indicated in the figure legend.

## Results

**Figure 1:**
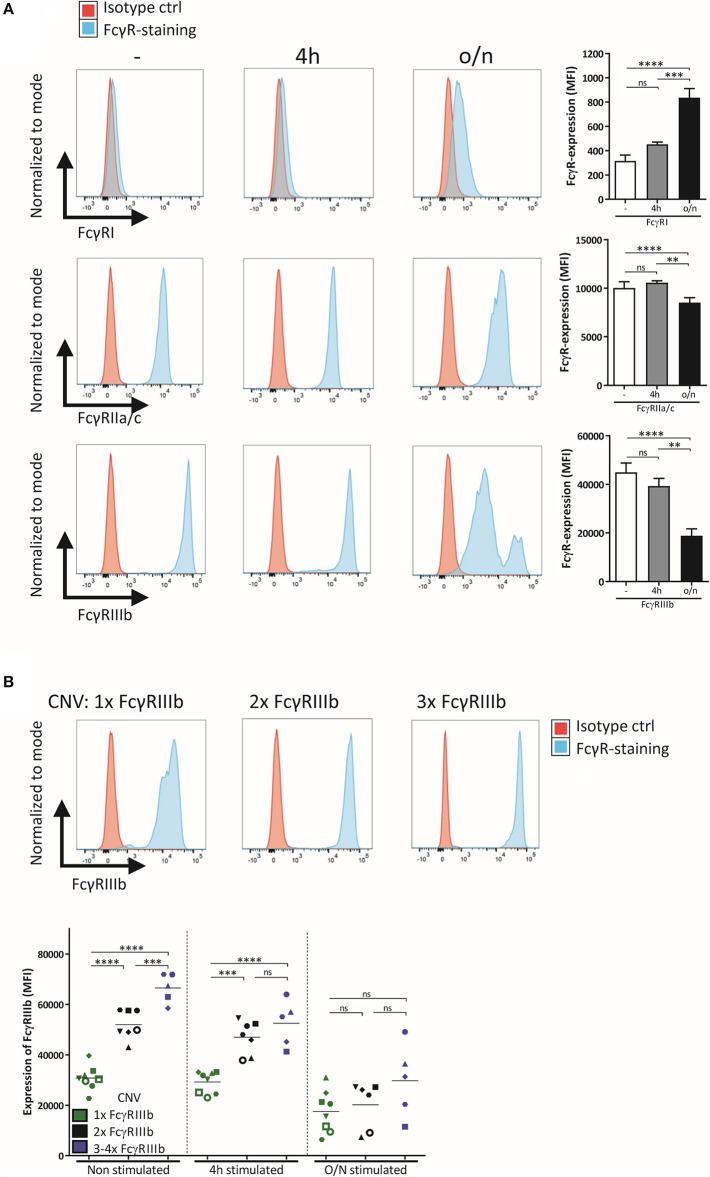
Expression of FcγR on neutrophils depends on activation status and CNV. **(A)** FcγR expression, shown as representative histograms and bar graphs (MFI), was determined for freshly isolated neutrophils and neutrophils stimulated for 4 h or overnight with G-CSF and IFNγ. **(B)** Neutrophils were isolated from donors with different copy numbers of *FCGR3B*, and their FcγRIIIb expression was checked using flow cytometry, before stimulation, after 4 h stimulation and after overnight stimulation with G-CSF and IFNγ. Individuals with only one copy of *FCGR3B* are represented by green dots, donors with two copies by black dots, donors with three or more copies of *FCGR3B* by blue dots. Each symbol represents an individual donor per color. Data shown are mean + SEM **(A)** and mean **(B)** with *N* = 20 **(A)** and *N* = 5–8 **(B)**, statistical analysis was performed by one-way paired ANOVA with Tukey *post-test*. ns, non-significant; ^**^p < 0.01, ^***^p < 0.001, and ^****^p < 0.0001.

We studied the role of FcγRIIIb during neutrophil ADCC toward solid cancer cells. Although FcγRIIIb is apparently unable to signal by itself, it definitely has the capacity to bind IgG and as such could potentially influence responses via other activating Fcγ-receptors on neutrophils, in either a positive or negative fashion. Such activating FcγRs present on neutrophils include FcγRI, only present after neutrophil activation, and FcγRIIa, which appears to be the main receptor required for ADCC against cancer cells expressing the tumor antigens HER2/Neu or EGFR (Supplementary Figure 1) (2, 3). Neutrophil-mediated ADCC toward cancer cells can be enhanced after neutrophil-activation, e.g., by granulocyte-colony stimulating factor (G-CSF) and interferon-γ (IFNγ) (32). This stimulation causes a change in the expression levels of the FcγRs, resulting in expression of FcγRI, a small decrease in expression in FcγRIIa, and, of particular interest, a substantial decrease in FcγRIIIb (Figure 1A). The reduction in FcγRIIIb expression could well be due to cleavage of FcγRIIIb by protease release after neutrophil activation (50, 51). As mentioned before FcγRIIIb is subject to considerable gene CNV (9), and the expression levels of FcγRIIIb are directly linked to the number of copies present in the genome (Figure 1B). Upon stimulation FcγRIIIb levels on neutrophils are gradually reduced and the variation among individuals with different FcγRIIIb levels are essentially blunted (Figure 1B).

### FcγRIIIb Functions as a Decoy Receptor During Neutrophil ADCC

**Figure 2:**
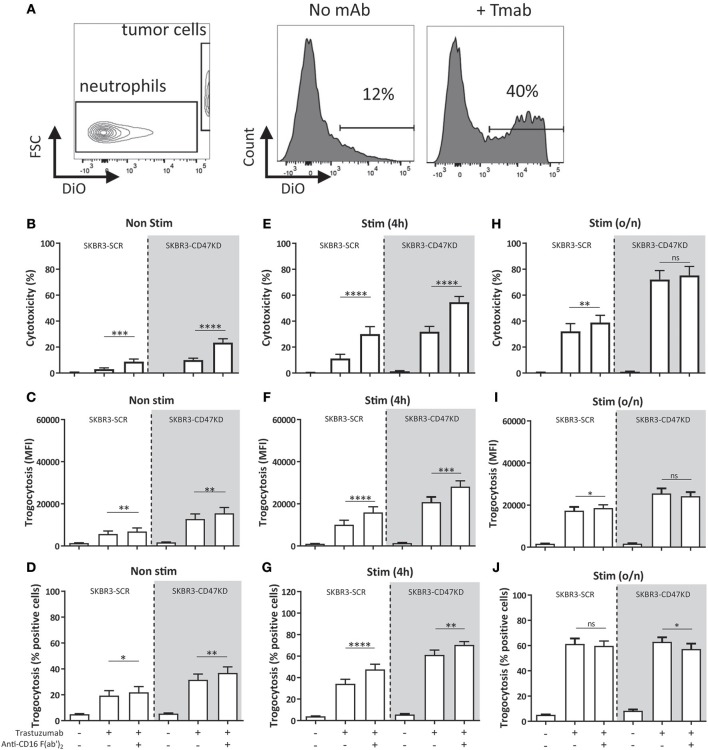
Inhibition of FcγRIIIb results in increased ADCC and trogocytosis. **(A)** Gating strategy for FACS-based trogocytosis assay. Histograms show neutrophil population becoming positive for DiO after incubation with trastuzumab coated SKBR3-scrambled (SKBR3-SCR) cells. **(B–J)** Blocking FcγRIIIb increases ADCC and trogocytosis of trastuzumab coated SKBR3-SCR cells (white background) when using non-stimulated **(B–D)**, 4 h stimulated **(E–G)** and to a lesser extent overnight **(H–J)** stimulated neutrophils (with G-CSF and IFNγ). This effect is also present when inhibiting CD47-SIRPα interactions by CD47 knock-down (SKBR3-CD47KD, gray background). Data shown are means + SEM with **(B)** *N* = 26, **(C)** *N* = 20, **(D)** *N* = 20, **(E)** *N* = 17, **(F)** *N* = 18, **(G)** *N* = 18, **(H)** *N* = 14, **(I)** *N* = 8, **(J)** *N* = 8, statistical analysis was performed by paired *t*-test. ns, non-significant; ^*^p < 0.05, ^**^p < 0.01, ^***^p < 0.001, and ^****^p < 0.0001.

We first determined the effect of blocking FcγRIIIb with F(ab')_2_ fragments of specific anti-FcγRIIIb on both their ability to take up cancer cell fragments (trogocytosis) (Figure 2A) as well as their cytotoxic capacity, as measured by ^51^Cr-release. The use of F(ab')_2_-fragments is absolutely critical here as intact anti-FcγR antibodies may also exert non-specific blocking by the so called “Kurlander” phenomenon (52). Blocking FcγRIIIb resulted in a prominent increase in both trogocytosis and ADCC, clearly suggesting that FcγRIIIb plays a negative role in neutrophil-mediated antibody-dependent destruction of cancer cells (Figures 2B–J). Similar results were obtained when using other solid cancer cells, such as the EGFR-positive A431 cell line combined with the therapeutic antibody cetuximab (Supplementary Figures 2A-C). However, with both tumor targets, this negative role of FcγRIIIb was only visible when using freshly isolated neutrophils or neutrophils that had only been briefly stimulated (i.e., for 4 h; Figures 2E–G), when relatively large quantities of FcγRIIIb are still present on the neutrophil cell surface (see Figure 1). In contrary, when evaluated after overnight stimulation with G-CSF and IFNγ both neutrophil trogocytosis and ADCC were higher and the effect of FcγRIIIb blocking eventually disappeared (Figures 2H–J), which could be explained, at least in part, by the observed reduction in FcγRIIIb surface expression (Figure 1). Of interest, under these conditions the enhancing effect of CD47-SIRPα interference on cytotoxicity was still clearly visible (Figure 2H). Whether or not FcγRIIIb is highly expressed on neutrophils, FcγRIIa remains the primary receptor responsible for triggering ADCC (Supplementary Figure 1). Thus, the principal FcγR mediating trogocytosis and subsequent ADCC of antibody-opsonized solid cancer cells by human neutrophils is FcγRIIa/c, and FcγRIIIb appears to function as a decoy receptor that apparently competes with FcγRIIa/c for binding to the Fc-portion of the opsonizing cancer therapeutic antibody.

To mimic checkpoint inhibitor blockade, we used SKBR3 cells with shRNA-knock-down for CD47 (reduction by ~85–90%) (SKBR3-CD47KD) to inhibit the interactions between CD47 and SIRPα (32, 33, 36). The effect of inhibiting both CD47-SIRPα interactions and FcγRIIIb became even more apparent (Figure 2, gray background), also indicating that disruption of CD47-SIRPα and FcγRIIIb blockade were not part of the same inhibitory pathway and that such interferences could generate additive effects.

We hypothesized that blocking of FcγRIIIb could perhaps be resulting in increased production of IL-8 by neutrophils, which was previously described to occur when crosslinking FcαRI on neutrophils (53). The use of IgA therapeutic antibodies enhances neutrophil-mediated ADCC of cancer cells compared to using IgG antibodies (54, 55), which could be in part due to the production of cytokines, such as IL-8, by the neutrophils. We therefore determined the presence of IL-8 in the supernatant after neutrophil-mediated ADCC of SKBR3 cells in the presence or absence of FcγRIIIb blocking antibodies. The IL-8 levels that were produced using an anti-HER2 IgG antibody were significantly lower compared to IgA, as reported before (45), and additional inhibition of FcγRIIIb showed no enhanced production of IL-8 (Supplementary Figure 2D).

### FCGR3B CNV Determines Neutrophil ADCC

**Figure 3:**
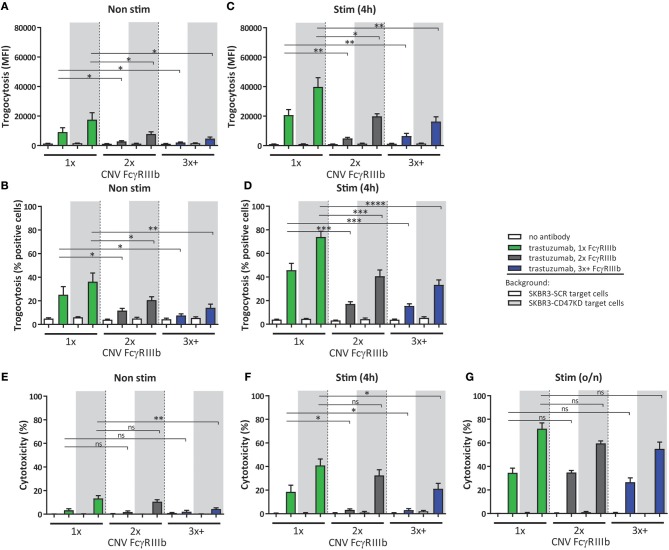
CNV of *FCGR3B* affects ADCC of cancer cells by neutrophils. Trogocytosis **(A–D)** and ADCC **(E–G)** was determined for neutrophils from donors with various copies of *FCGR3B*. Freshly isolated **(A,B,E)**, 4 h stimulated **(C,D,F)** or overnight stimulated **(G)** neutrophils were combined with trastuzumab coated SKBR3-SCR (white background) or SKBR3-CD47KD cells (gray background). Shown are results from individuals with one copy (green), two copies (gray), or three or more copies (blue) of *FCGR3B*. Data shown are means + SEM with results from multiple experiments with donors ranging from *N* = 7–10. Statistical analysis was performed by one-way paired ANOVA with Sidak *post-test*. ns, non-significant; ^*^p < 0.05, ^**^p < 0.01, ^***^p < 0.001, and ^****^p < 0.0001.

As indicated above FcγRIIIb surface expression on neutrophils is subject to considerable variation, and this is largely caused by gene copy number variation within the *FCGR2/3* locus (15). This enabled us to further study the observed negative contribution of FcγRIIIb to neutrophil ADCC. We therefore evaluated neutrophils from individuals with different copy numbers of *FCGR3B* determined by MPLA-based genotyping (49). Indeed, individuals with one copy of the gene have significantly increased ADCC and trogocytosis capacity compared to individuals with 2 or 3 or more copies, using neutrophils either freshly isolated or after 4 h stimulation with G-CSF and IFNγ (Figure 3). However, after overnight stimulation this difference essentially disappeared in all tested individuals and irrespective of *FCGR3* gene copy number (Figure 1). When comparing individuals with low (1x) and high FcγRIIIb (2–4x) expression blocking of FcγRIIIb could enhance ADCC to indistinguishable levels (Supplementary Figure 3), demonstrating that the difference in ADCC capacity between individuals with different *FCGR3B* CNV can indeed largely be attributed to the difference in FcγRIIIb expression on neutrophils. In these experiments the levels of other surface molecules relevant in the context of neutrophil ADCC (31, 32), including FcγRs, integrins or SIRPα were similar in all donors with different copy numbers of *FCGR3B* (Supplementary Figure 4).

When correlating the FcγRIIIb expression to either trogocytosis or ADCC capacity of neutrophils, irrespective of *FCGR3B* genetic status, we also noted a significant inverse correlation, but as expected this occurred only when using either freshly isolated neutrophils (Supplementary Figures 5A-C) or 4 h (Supplementary Figures 5D-F) stimulated neutrophils, but this correlation disappeared upon overnight neutrophil stimulation (Supplementary Figures 5G-I) consistent with the loss of surface FcγRIIIb. By comparison, we did not find any significant correlations when comparing FcγRIIa expression levels and killing (Supplementary Figure 6). These findings show that in non-stimulated neutrophils *FCGR3B* CNV is an important determinant of ADCC capacity, with higher levels of CNV and concurrent FcγRIIIb surface expression negatively affecting neutrophil ADCC, thereby providing genetic evidence for a role of FcγRIIIb as a decoy receptor.

### Antibody Afucosylation Negatively Impacts Neutrophil ADCC

**Figure 4:**
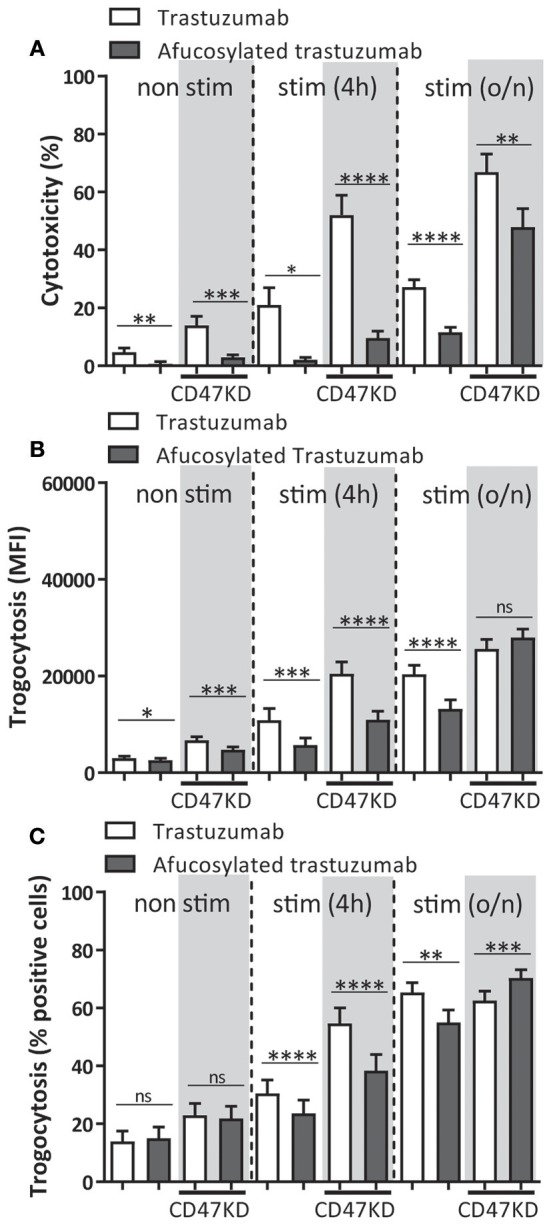
Neutrophil ADCC and trogocytosis are reduced when afucosylated therapeutic antibodies are used. Afucosylated trastuzumab was compared to regularly fucosylated trastuzumab in both trogocytosis **(A,B)**, and ADCC **(C)**. Freshly isolated, 4 h and overnight stimulated (with G-CSF and IFNγ) neutrophils were combined with IgG opsonized SKBR3-SCR (white background) or SKBR3-CD47KD cells (gray background). Data shown are means + SEM with results from multiple experiments with donors ranging from *N* = 9–16. Statistical analysis was performed by paired *t*-test. ns, non-significant; ^*^p < 0.05, ^**^p < 0.01, ^***^p < 0.001, and ^****^p < 0.0001.

A number of mutations and posttranslational modifications of therapeutic antibodies have previously been explored for the purpose of improving their clinical potential. One of these alterations is antibody afucosylation, which changes the glycan linked to asparagine at position 297 (N297). Afucosylation of this glycan increases the binding affinity of the antibody to FcγRIIIa (44, 56, 57), and this has been shown to increase ADCC by PBMC, including NK cells and monocytes, that express activating FcγRIIIa (58–61). However, afucosylation also improves binding to FcγRIIIb ~15 fold (44) compared to normal IgG which impacts neutrophil ADCC (2, 62), but to what extend this affects neutrophil trogoptosis toward tumor cells has not been previously investigated. Consistent with the above findings, neutrophil-mediated ADCC of SKBR3 cells using afucosylated trastuzumab resulted in a highly significant and prominent (up to ~80–90%) decrease in ADCC when compared to normally fucosylated trastuzumab (Figure 4A). Interestingly, trogocytosis was also substantially affected and showed both a decrease in the net-amount of target membrane uptake on average by neutrophils (Figure 4B) and decrease in the number of participating neutrophils (Figure 4C), confirming the negative effect of FcγRIIIb under these conditions. As expected from the above the difference in ADCC response between afucosylated and fucosylated trastuzumab became smaller when neutrophils had been activated. Furthermore, by inhibiting FcγRIIIb on neutrophils we were able to completely rescue the ability of afucosylated trastuzumab to perform ADCC and trogocytosis (Supplementary Figure 7) showing that the reduced killing of afucosylated trastuzumab by neutrophils can indeed be entirely attributed to its enhanced binding to FcγRIIIb. Clearly, this shows that antibody afucosylation, while enhancing the ADCC capacity of NK cells and monocytes, negatively affects neutrophil ADCC.

### FcγRIIIb Contributes to IgG-Mediated Phagocytosis of Bacteria

**Figure 5:**
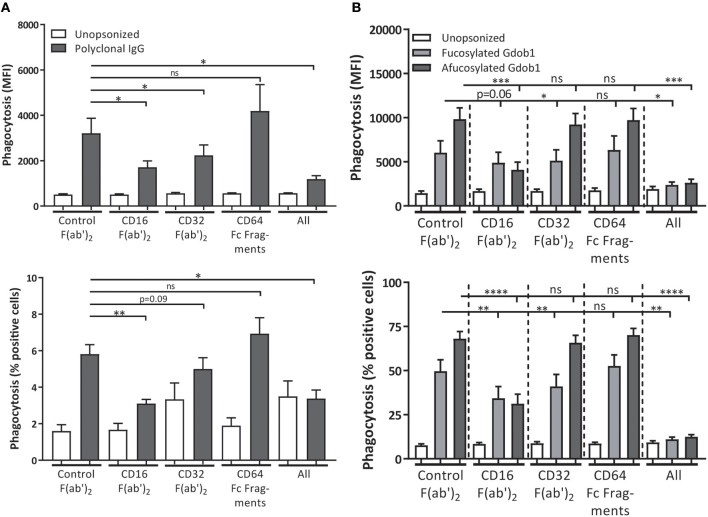
FcγRIIa and FcγRIIIb both contribute to bacterial phagocytosis. FcγRs were blocked on freshly isolated neutrophils during phagocytosis of polyclonal IgG-opsonized *S. aureus* (gray bars) **(A)** or heat-killed *S. pneumoniae*, serogroup 6B, opsonized with GDob1 (IgG_1_) (dark gray bars) or afucosylated GDob1 (IgG_1_) (light gray bars) **(B)**. Shown are both percentage of neutrophils phagocytosing (% positive cells) and relative uptake of bacteria (MFI). Data shown are means + SEM with results shown from 3 **(A)**, and 2 **(B)** experiments with **(A)** *N* = 10, **(B)** *N* = 7, statistical analysis was performed by one-way paired ANOVA with Dunnett's *post-test*. ns, non-significant; ^*^p < 0.05, ^**^p < 0.01, ^***^p < 0.001, and ^****^p < 0.0001.

It has previously been shown that FcγRIIIb does stimulate phagocytosis of bacteria and platelets cooperatively with other activating FcγRs, such as FcγRIIa, which is further stimulated by afucosylation of the opsonizing antibodies (7, 38, 40). To determine whether we could replicate this cooperative role we used *S. aureus* opsonized with polyclonal human IgG, which is a commercial blood product containing polyclonal IgG isolated and pooled from thousands of donors. We noticed that blocking either FcγRIIa or FcγRIIIb on neutrophils resulted in a decreased phagocytosis of *S. aureus*, with the most optimal reduction in phagocytosis being achieved by blocking both receptors (Figure 5A). No role for FcγRI in bacterial phagocytosis by neutrophils was found. However, since polyclonal IgG contains all IgG isotypes (approx. 65% IgG_1_) and our ADCC experiments are done using only monoclonal IgG_1_ antibodies we wanted to be certain that these results were not due to effects of one of the other IgG isotypes. To be able to specifically look at IgG_1_ mediated effects, we used a heat-killed *Streptococcus pneumoniae* of serogroup 6B, which can be opsonized with a 6B-specific recombinant human IgG1 monoclonal antibody (GDob1) (47). This confirmed a cooperative role of FcγRIIa and FcγRIIIb, with the two receptors functioning in a largely redundant fashion with no additive role for FcγRI (Figure 5B; Supplementary Figure 8). Of interest, when using an afucosylated variant of GDOb1, with increased affinity for FcγRIIIb, FcγRIIIb clearly became the dominant FcγR mediating phagocytosis (Figure 5B). Collectively, this corroborates previous results that FcγRIIIb on human neutrophils plays a facilitating role in microbial phagocytosis, and this strongly contrasts with the negative role of this receptor during ADCC.

## Discussion

**Figure 6:**
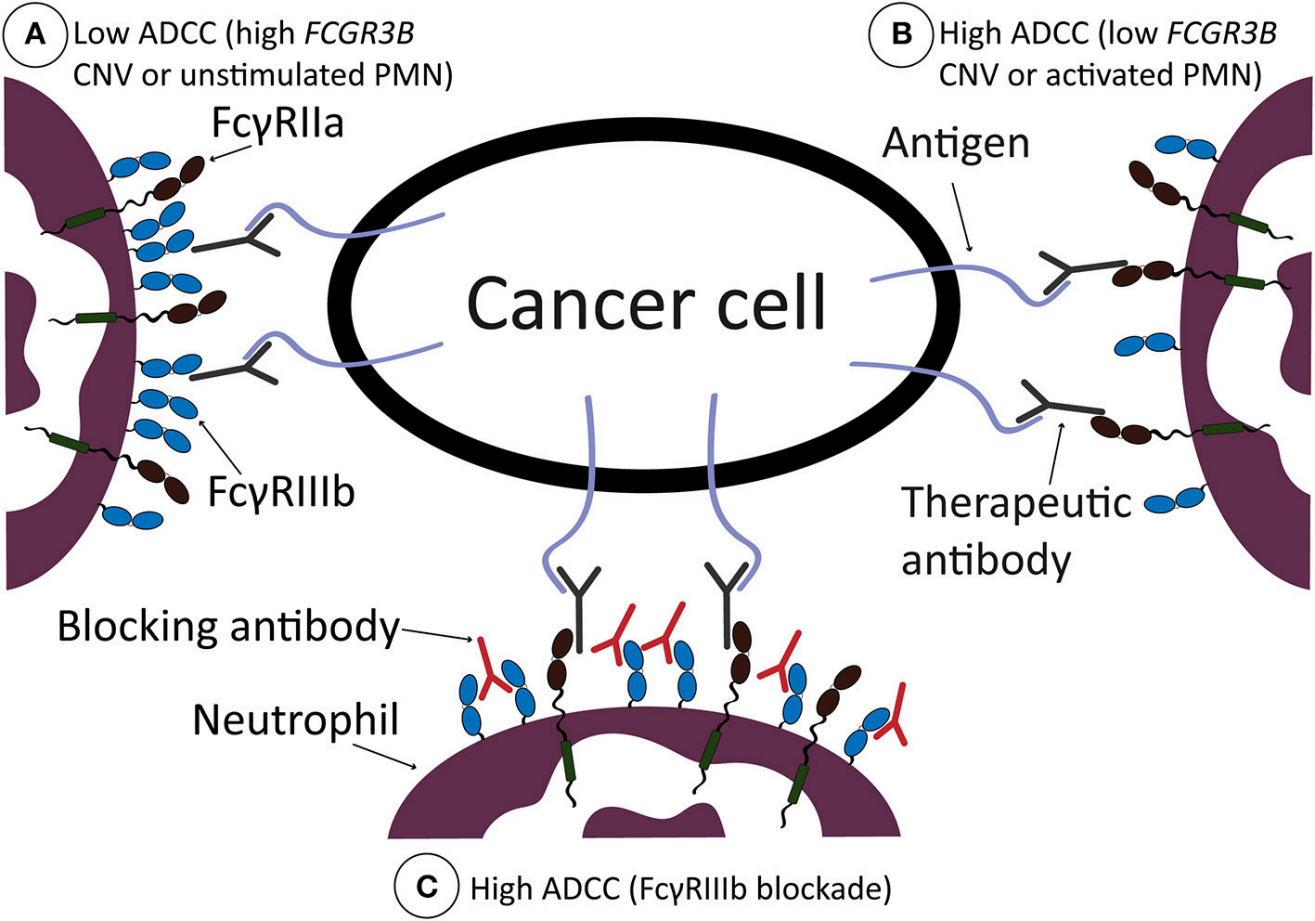
Graphical representation of working model. ADCC by neutrophils is low when neutrophils are not stimulated, or if there is a high number of FcγRIIIb present on the neutrophil cell surface, due to high CNV of *FCGR3B* **(A)**. ADCC is high when neutrophils are stimulated, with G-CSF and IFNγ, or when there is a low number of FcγRIIIb present on the neutrophil cell surface, due to low CNV of *FCGR3B* **(B)**. ADCC by neutrophils can be increased after therapeutic intervention, i.e., by blocking FcγRIIIb with blocking antibodies, which results in high ADCC **(C)**. In all situations, FcγRIIa is required for neutrophil ADCC and is, due to high presence of FcγRIIIb in **(A)**, unable to sufficiently bind the therapeutic antibody opsonizing the cancer cell surface. When there is less FcγRIIIb present on the cell surface **(B)** or after FcγRIIIb blockade **(C)**, neutrophils are more effective in ADCC of solid cancer cells.

Here we found that FcγRIIIb on neutrophils acts as a decoy receptor during human neutrophil ADCC toward cancer cells, thereby restricting tumor killing mechanisms exerted via FcγRIIa. This is in line with previous reports showing that signaling through FcγRIIa is apparently entirely essential for active ADCC in neutrophils (2, 3). For phagocytosis, other mechanisms are apparently at play, as we and others found neutrophil FcγRIIIb to actively participate in bacterial ingestion (7, 63). This is possible as FcγRIIIb is a GPI-linked receptor, causing it to preferentially reside in detergent-resistant membranes, or lipid rafts, enriched in signaling molecules such as myristoylated src-kinases. In addition, it associates through its ectodomains with other receptors, and certainly with other FcγR in *cis* during encounter with IgG-opsonized targets, providing receptor cross-talk (4, 39, 64, 65). The enhanced recognition via FcγRIIIb apparently facilitates phagocytosis, while in contrary it impedes ADCC as we show here. Of interest, this may not only be true for phagocytosis of microbes and/or small particles, but maybe also for relatively small tumor cells such as CLL cells. Here, FcγRIIIb seems to have a beneficial effect (38, 66), although there still seems to be some discussion about whether small tumor cells are phagocytosed or in fact trogocytosed by human neutrophils (67). In general, antibodies of the IgG1 subclass bind to the various Fcγ-receptors expressed on neutrophils with a wide range of affinities. In particular, the binding affinity of FcγRIIIB for IgG1 is approximately 10-fold lower compared to FcγRIIA (68). This might explain the relative high amount of FcγRIIIB molecules on the neutrophil plasma membrane needed to create the “buffering” decoy effect of FcγRIIIB as we describe herein in the context of ADCC specifically (see Figure 6 for a graphical representation).

In further support that FcγRIIIb negatively affects ADCC, we found a clear gene-dosage effect of *FCGR3B* through the CNV of the gene, with higher numbers gradually decreasing ADCC even further. Potentially *FCGR3B* CNV can be used as a new biomarker for cancer immunotherapy, where patients can be stratified with likelihood of benefitting from therapy when patients have a lower *FCGR3B* CNV combined with the right tumor antigens (e.g., HER2/Neu or EGFR).

To date, this FcγRIIIb decoy effect during ADCC has not been possible to study *in vivo* due to the fact that mice do not express a GPI-linked FcγR ortholog or homolog (27–30). However, in the future it might be interesting to study this effect in humanized models [mice expressing human FcγR or mice with human immune system (69)] to see the relative contribution of FcγRIIIb on neutrophils in therapy and if its effect can be circumvented.

Furthermore, our findings raise doubt whether the use of afucosylated monoclonal antibodies for antibody therapy against cancer is beneficial in all situations. Glycoengineering antibodies in this manner is currently being applied to various monoclonal antibodies to increase their capability to enhance ADCC and phagocytosis. This modification is well-documented to increase binding to FcγRIIIa, which is expressed by natural killer cells, monocytes and macrophages, (62, 70, 71). Less consideration has been given to the fact that this type of glycoengineering similarly enhances its affinity to FcγRIIIb, which is only present on granulocytes (44). Here, we confirm that engineered antibodies with enhanced affinity to FcγRIIIb by afucosylation have deleterious effects on ADCC by neutrophils (2, 62). This effect could partially be negated by using a combination of a targeting antibody and preventing the CD47-SIRPα- checkpoint inhibitor axis. Thus, it can be anticipated that the net effect of cancer therapeutic antibody afucosylation is basically a trade-off between the beneficial effects on various immune cells on one hand and the detrimental effects on neutrophils.

One of the obvious implications of our findings is that selective blockade of FcγRIIIb could be a potential way to enhance the effect of cancer therapeutic antibodies and thereby improve clinical outcome for patients and/or reduce their need for other non-specific agents such as chemotherapeutics. However, while interesting to explore further this is not a trivial challenge as the activating FcγRIIIa receptor on other cells has a very similar extracellular region, making it perhaps impossible to achieve the required specificity. Nevertheless, as we show here the effects of blocking FcγRIIIb appear interesting so if the issue of specificity can be solved one way or another this may be an interesting concept to pursue (see also Figure 6 for a graphical representation).

We have shown in the current study that inhibition of FcγRIIIb also increases ADCC when this is combined with interference of CD47-SIRPα interactions. FcγRIIIb specific inhibiting agents could thus potentially be combined with antibodies targeting these checkpoint-inhibitor molecules, which are currently in development (www.clinicaltrials.gov identifiers: NCT02216409; NCT02678338, NCT02641002; NCT02367196, NCT02890368; NCT02663518, NCT02953509) (72). In theory, using a monoclonal antibody with an increased affinity to FcγRIIa (2) could also be beneficial to circumvent the decoy effect by FcγRIIIb.

Collectively, we have shown that FcγRIIIb acts as a decoy receptor for IgG during neutrophil-mediated ADCC of solid cancer cells, while it harbors a good potential to stimulate phagocytosis. These results pinpoint FcγRIIIb as a potential target and biomarker for cancer immunotherapy, while underscoring a potential threat using glycoengineered antibodies with enhanced binding to both FcγRIIIa and FcγRIIIb which needs to be further evaluated in patients.

## Author Contributions

LT, MvH, MH, HM, KF, RvB, TK, MvE, GV, and TvdB designed research. LT, MvH, CB, MH, XZ, JvdH, SN, PV, and JG performed research. SL-T, TV, MP, and GV contributed new reagents analytic tools. LT analyzed data. LT, HM, RvB, and TvdB wrote the paper that was edited and approved by all authors.

### Conflict of Interest Statement

The authors declare that the research was conducted in the absence of any commercial or financial relationships that could be construed as a potential conflict of interest.

## Abbreviations

FcγR: Fcγ receptor
ADCC: antibody dependent cellular cytotoxicity
NK cell: natural killer cell
ADCP: antibody dependent cellular phagocytosis
ITAM: immunoreceptor tyrosine-based activation motif
CNV: copy number variation
G-CSF: granulocyte-colony stimulating factor
IFNγ: interferon-γ.
