# Supplementary material for: FcγRIIIb Restricts Antibody-Dependent Destruction of Cancer Cells by Human Neutrophils
Source: Front Immunol. 2019 Jan 30;9:3124. doi: 10.3389/fimmu.2018.03124 (PMC6363688; doi:10.3389/fimmu.2018.03124)
Supplement: Supplementary file 1 [file Data_Sheet_1.PDF]

S1

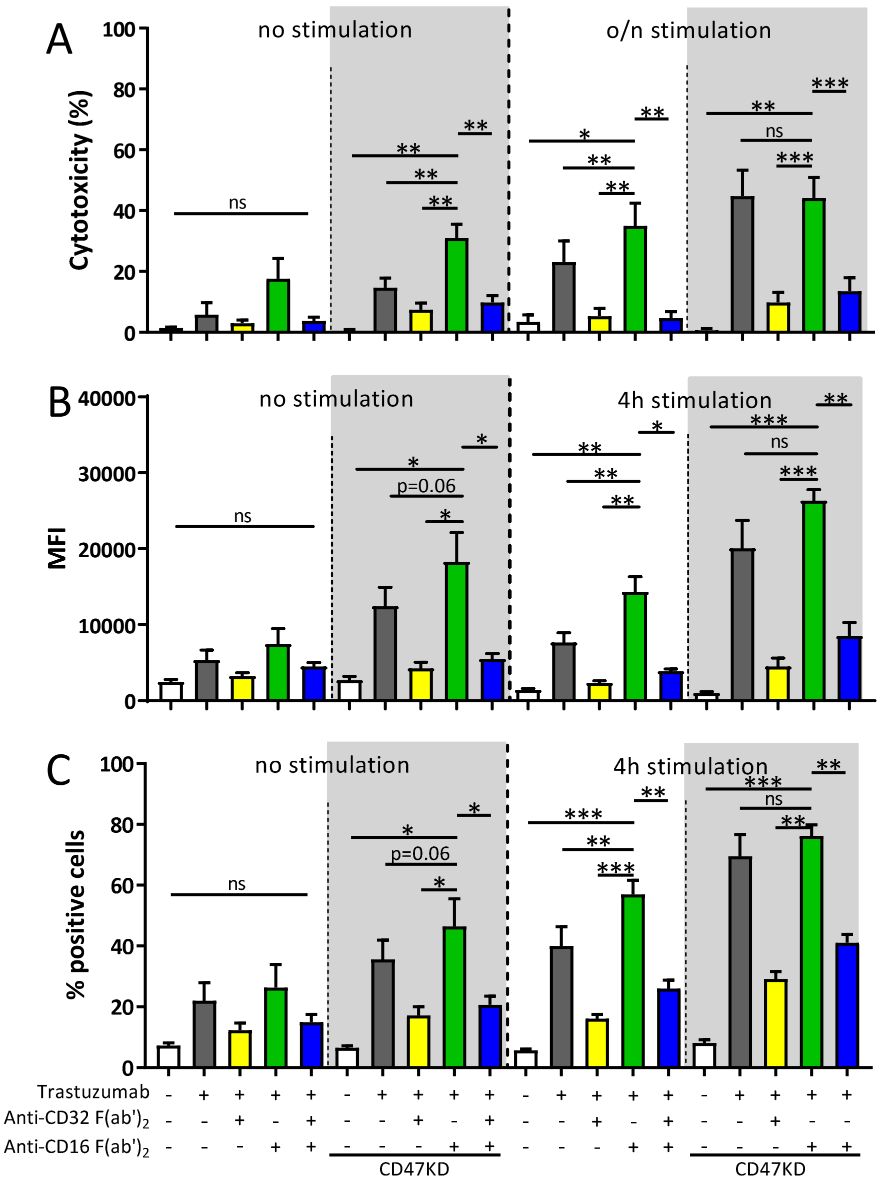

Supplementary Figure 1. FcγRIIa is required for ADCC of cancer cells

FcγRs were blocked on non-stimulated, overnight stimulated (ADCC) or 4h stimulated (trogocytosis) neutrophils during ADCC (A) or trogocytosis (B,C) of trastuzumab coated SKBR3-SCR (white background) or SKBR3-CD47KD (grey background) cancer cells. Stimulation using G-CSF and IFNγ. Data shown are means + SEM with results from 3 experiments with a total of donors ranging from N=7-89. Statistical analysis was performed by one-way paired ANOVA with Dunnett's post test. ns= non-significant, *p < 0.05, **p < 0.01, and ***p < 0.001.

S2

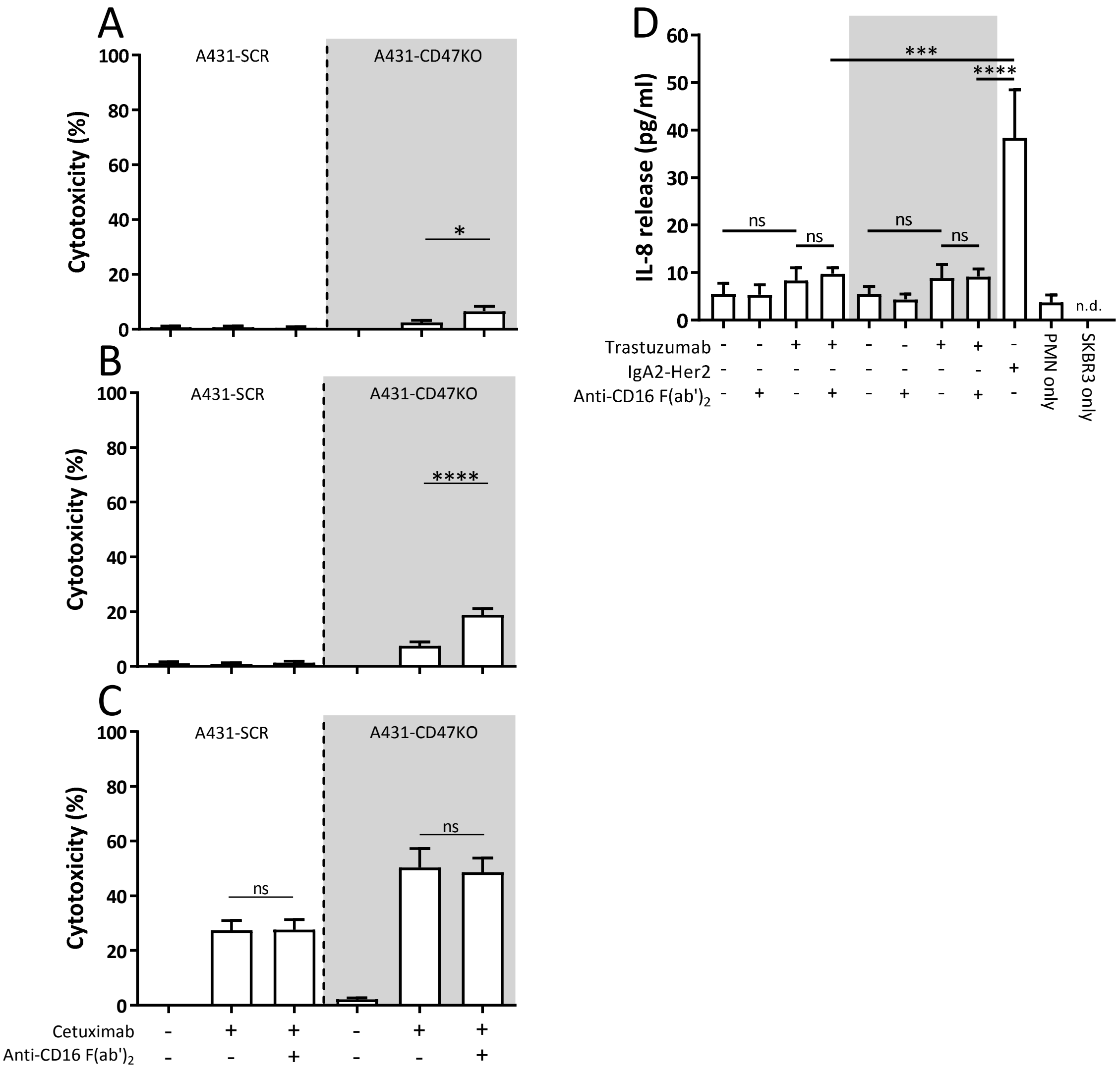

Supplementary Figure 2. Inhibition of FcγRIIIb results in increased ADCC, but not increased IL-8 production

Blocking FcγRIIIb during ADCC of cetuximab coated A431 (white background) or A431-CD47KO cells using non-stimulated neutrophils (A), 4h stimulated neutrophils (B), or overnight stimulated neutrophils (C) (stimulation with G-CSF and IFNγ). (D) IL-8 production during ADCC of SKBR3 cancer or SKBR3-CD47KD (grey background) cells by neutrophils, determined using ELISA. Anti-HER2-IgA2 is used as a positive control. Data shown are means + SEM with results from (A) N=9, (B) N=11, (C) N=6, (D) N=4 individual donors. Statistical analysis was performed by paired t-test (A-C) or paired one way ANOVA (D) with Sidak's post test. ns= non-significant, *$p < 0.05$, ***$p < 0.001$, ****$p < 0.0001$, and n.d= not detected.

S3

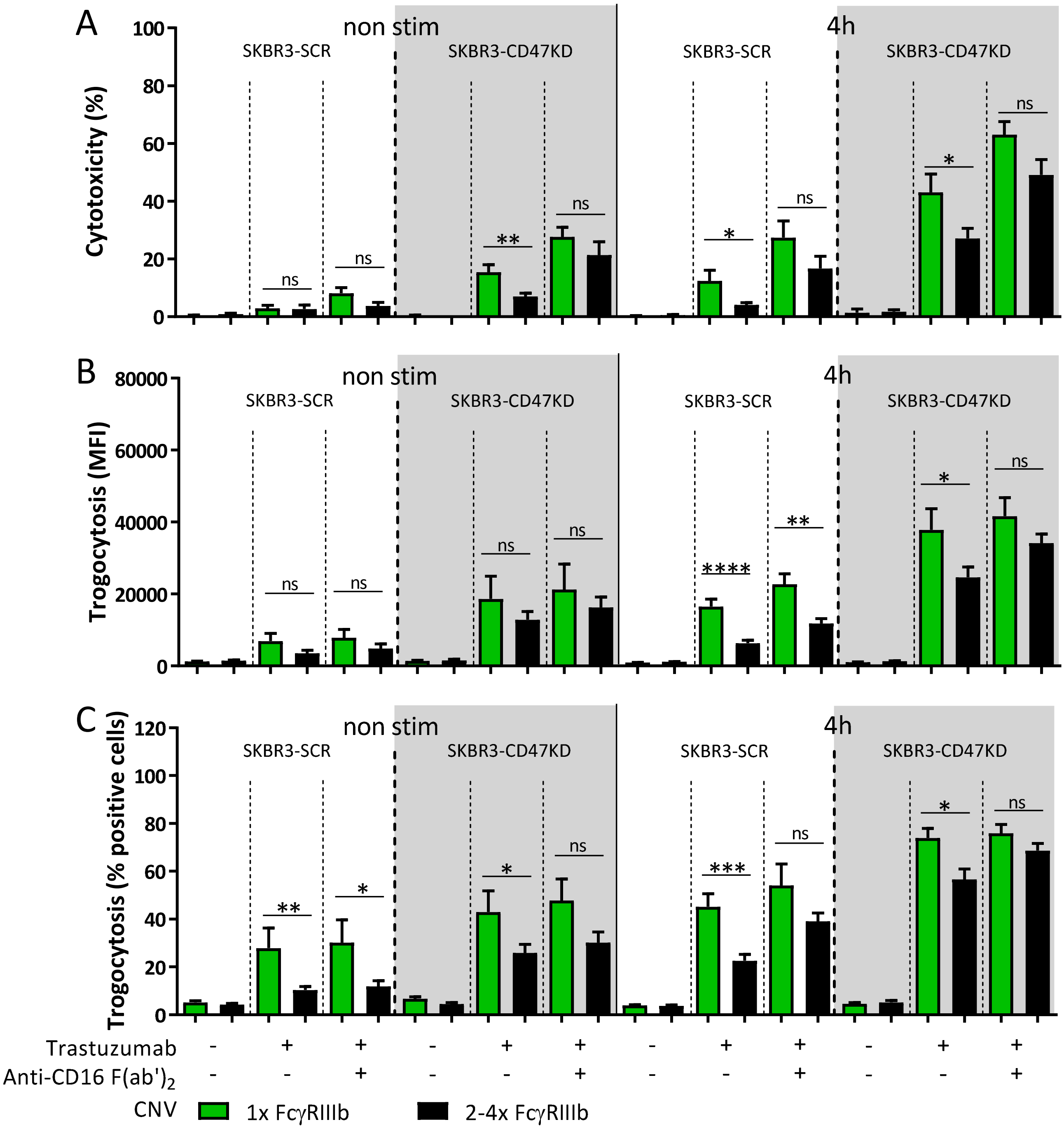

Supplementary Figure 3. Inhibition of FcγRIIIb rescues ADCC by neutrophils of donors with higher CNV

FcγRIIIb was blocked on non-stimulated or 4h stimulated (with G-CSF and IFNγ) neutrophils during ADCC (A) and trogocytosis (B,C) of SKBR3-SCR (white background) and SKBR3-CD47KD (grey background) cells. Data shown are means + SEM with results from multiple experiments with donors ranging from N=6-15. Statistical analysis was performed by paired t-test. ns= non-significant, *$p < 0.05$, **$p < 0.01$, ***$p < 0.001$, and ****$p < 0.0001$.

S4

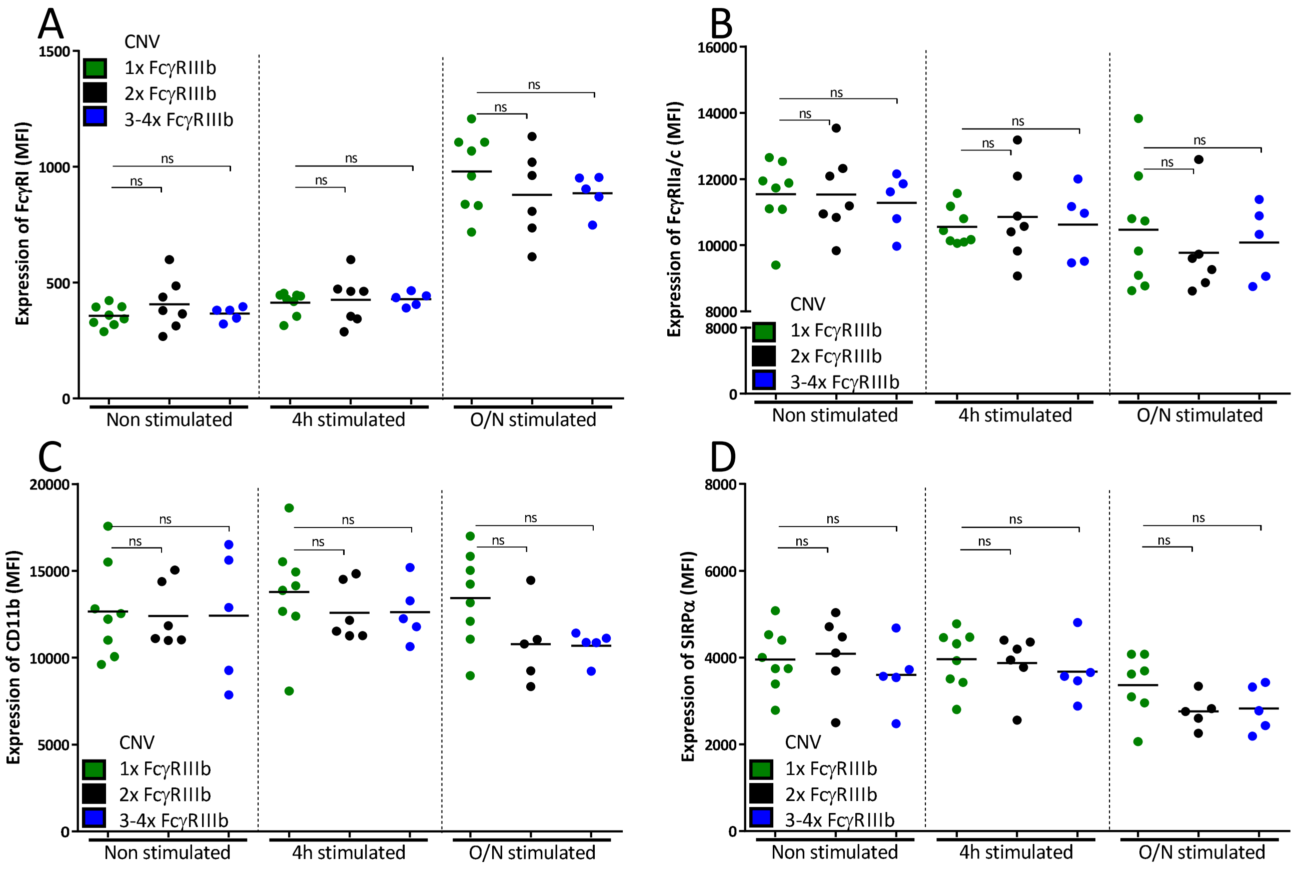

Supplementary Figure 4. Enhanced ADCC and trogocytosis is not due to other FcγRs, integrins or SIRPα

Expression of FcγRI (A), FcγRIIa (B), CD11b (C), and SIRPα (D) were determined using flow cytometry for donors having various copies of FCGR3B. Shown are donors with one copy (green dots), two copies (black dots) or three or more copies (blue dots). Data shown are means with results from multiple experiments with donors ranging from N=5-8. Statistical analysis was performed by one-way paired ANOVA with Dunnett's post test.

S5

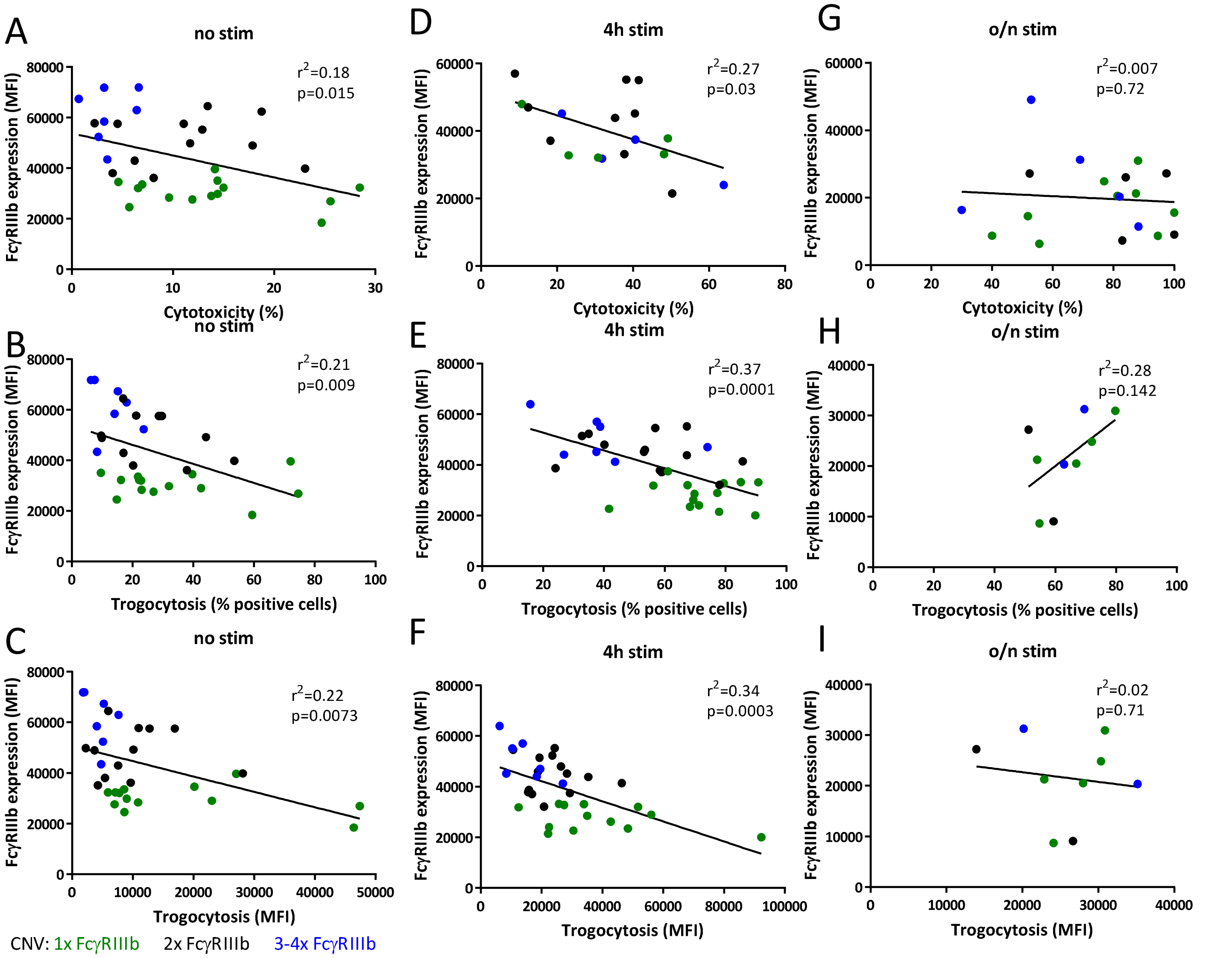

Supplementary Figure 5. Significant correlation between ADCC and trogocytosis capacity of neutrophils and FcγRIIIb expression

ADCC (A,D,G) and trogocytosis (B,C,E,F,H,I) levels were correlated to FcγRIIIb expression on neutrophils. Significance is determined by a linear regression line through data points. Visualized are donors with 1 copy (green dots), 2 copies (black dots) or 3 or more copies (blue dots) of FCGR3B.

S6

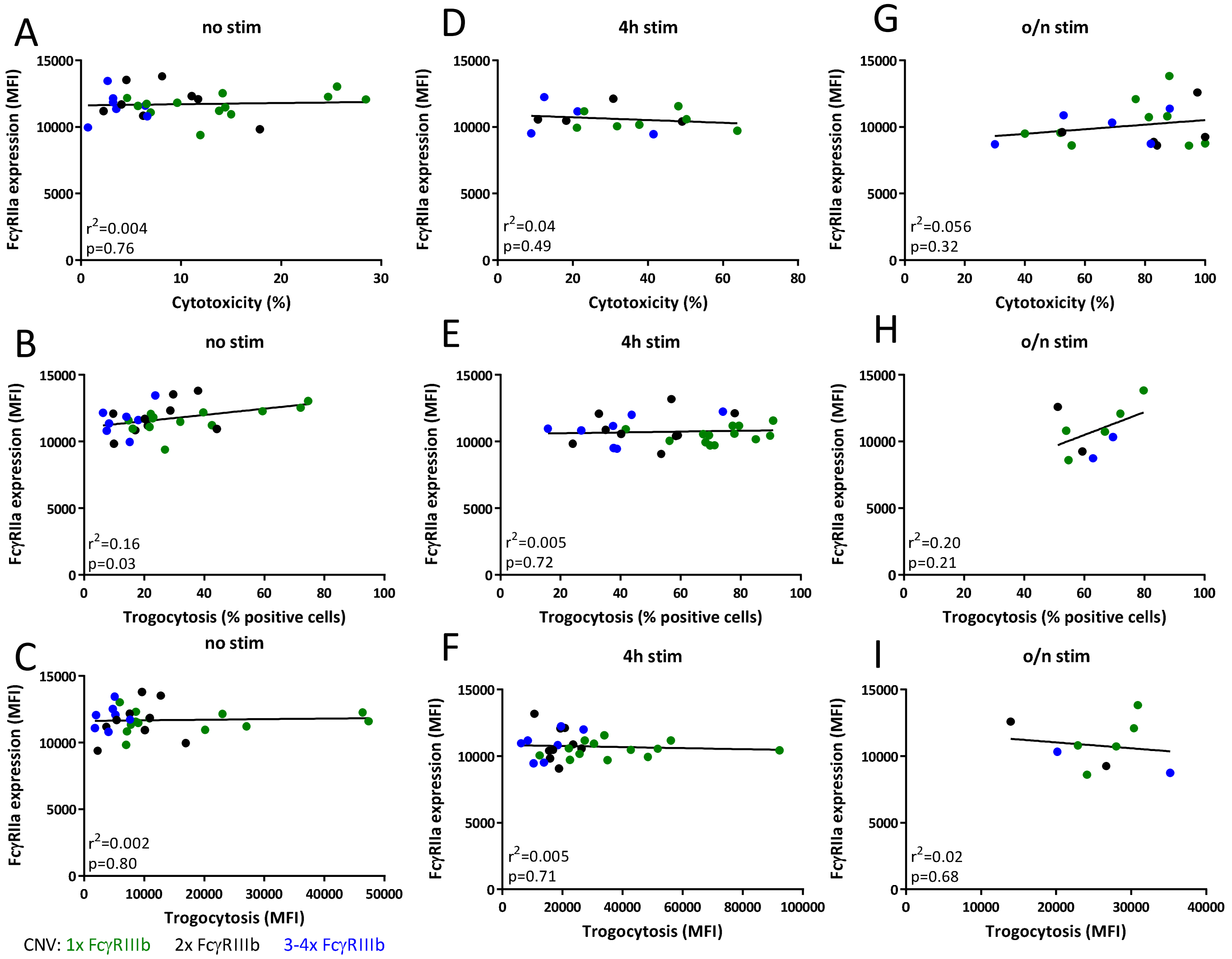

Supplementary Figure 6. No significant correlation between ADCC and trogocytosis capacity of neutrophils and FcγRIIa expression

ADCC (A,D,G) and trogocytosis (B,C,E,F,H,I) levels were correlated to FcγRIIa expression on neutrophils. Significance is determined by a linear regression line through data points. Visualized are donors with 1 copy (green dots), 2 copies (black dots) or 3 or more copies (blue dots) of FCGR3B.

S7

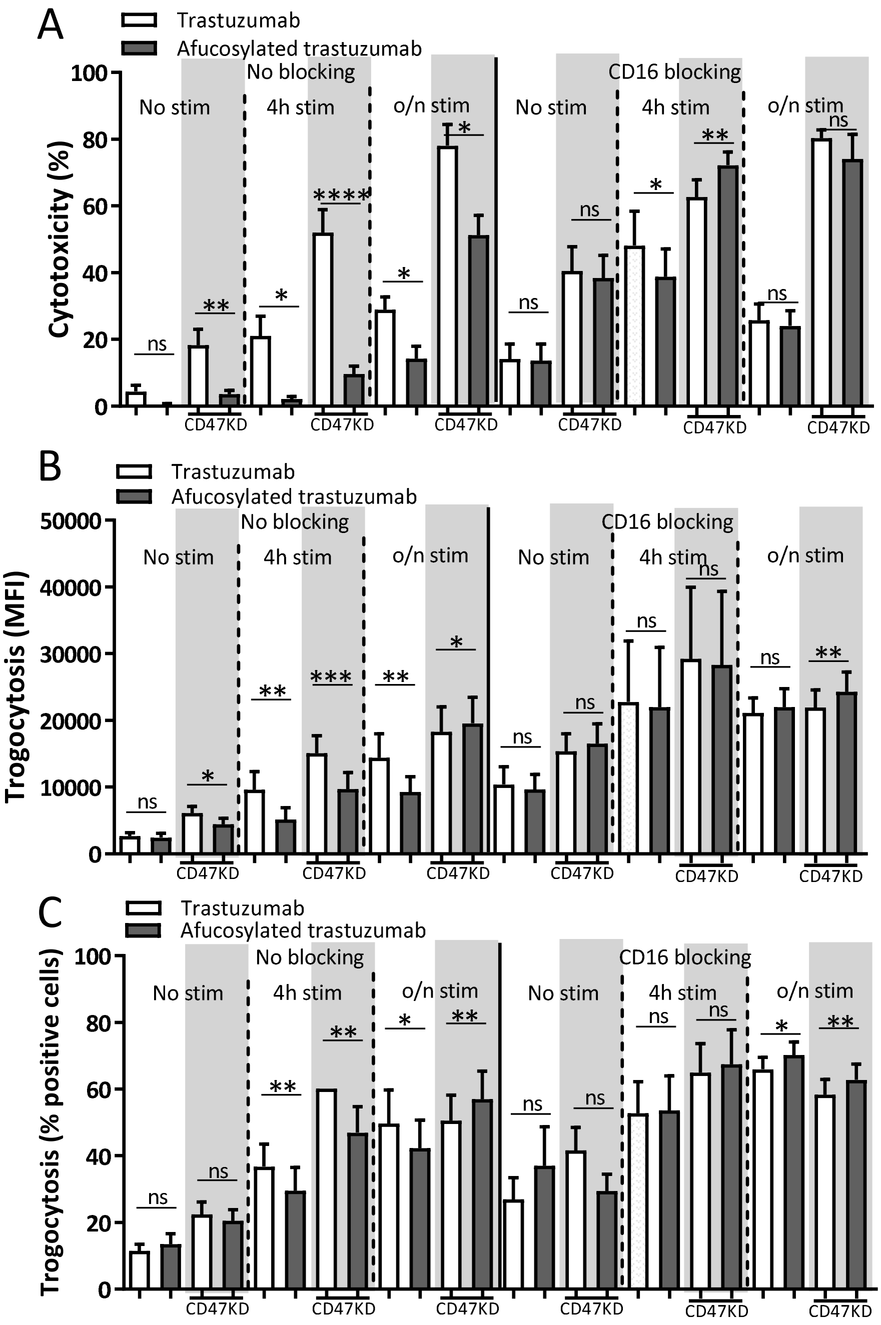

Supplementary Figure 7. Negative effect of using afucosylated trastuzumab can be reversed after FcγRIIIb inhibition.

ADCC (A) and trogocytosis (B,C) of SKBR3-SCR (white background) and SKBR3-CD47KD (grey background) cancer cells by neutrophils using normal (white bars) and afucosylated (grey bars) trastuzumab. On the right side of the graph FcγRIIIb was inhibited on neutrophils. Data shown are means + SEM with results from 6-9 individual donors from 3-4 separate experiments. Statistical analysis was performed by paired t-test. ns= non-significant, *p < 0.05, **p < 0.01, ***p < 0.001, and ****p < 0.0001.

S8

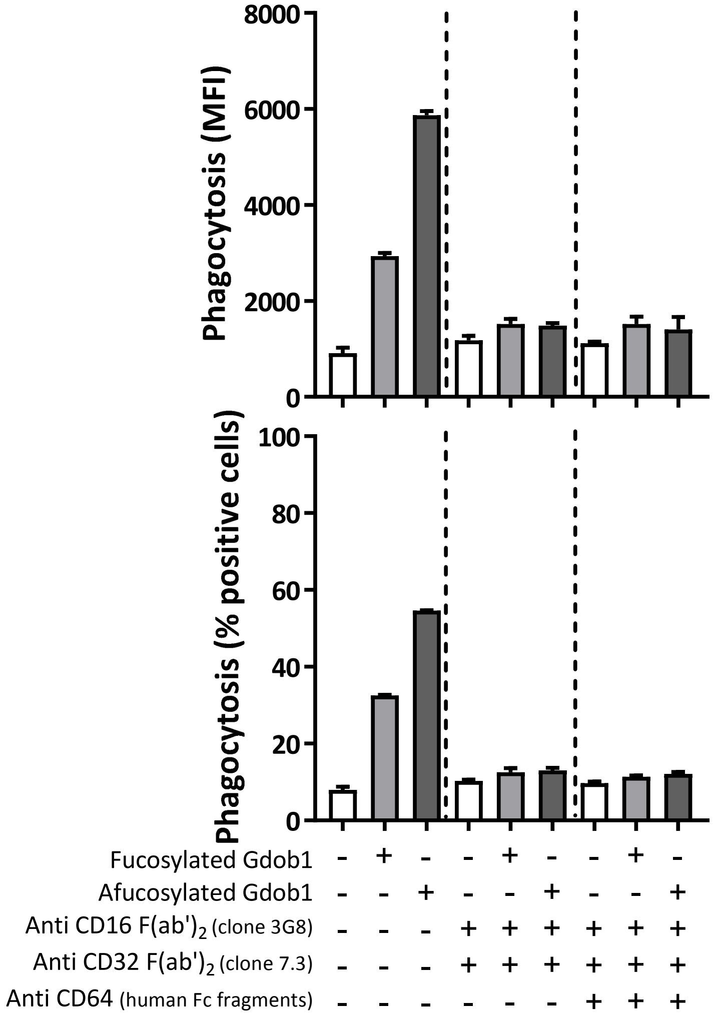

Supplementary Figure 8. FcγRI does not contribute to phagocytosis of S. pneumoniae

FcγRs were blocked on freshly isolated neutrophils during phagocytosis of heat-killed S. pneumoniae, serogroup 6B, opsonized with fucoslyated- or afucosylated GDob1 (IgG1). Data shown are means + SEM and are from one representable experiment with N=2.
